# Point-of-care ultrasound is useful for the detection of proximal ureteral stone in the emergency department

**DOI:** 10.1186/2036-7902-7-S1-A21

**Published:** 2015-03-09

**Authors:** Kuo-Chih Chen, Chee-Fah Chong, Tzong-Luen Wang

**Affiliations:** 1 Emergency Department, Shin Kong Wu Ho-Su Memorial Hospital, Taipei, Taiwan; School of Medicine, Fu-Jen Catholic University, New Taipei City, Taiwan

## Background

Point-of care ultrasound has many applications in the emergency department and one of the major applications is to detect hydronephrosis. For ureteral stones, emergency physicians use plain films, intra-venous urography and/or computed tomography as imaging modalities. The role of ultrasound for detection of ureteral stones remains uncertain.

## Objective

To assess the efficacy of point-of-care ultrasound for the detection of ureteral stone in the emergency department.

## Patients and methods

From January 1^st^ to June 30^th^ 2014, convenient patients presenting with hydronephrosis were scanned for the presence of ureteral stone in the emergency department of one tertiary medical center in northern Taiwan. One experienced emergency attending physician was responsible for the detection of ureteral stone using point-of-care ultrasound. We defined ureteral stones detected from the uretero-pelvic junction to iliac vessels as proximal ureteral stone and those from iliac vessels to uretero-vesicular junction as distal ureteral stone. The presence of ureteral stone were further confirmed by the combination of plain film, intravenous urography, computed tomography or the absence of hydronephrosis on followed sonography within 2 days.

## Results

Thirty-four patients were enrolled in this study and 19 patients were male. The mean age was 47, ranging from 29 to 71. Moderate to severe flank pain was the major reason for emergency visits and for detection of hydronephrosis in 82% of patients and the rest were scanned because of severe sepsis. Fifteen patients were found to have proximal ureteral stone. The detection rates for ureteral stones on ultrasound and plain film were 100% and 86.7%. For distal ureteral stone, the detection rate on ultrasound and plain film were 42% and 36.8%. Overall, the detection rates for ureteral stone on ultrasound, plain film and combination of ultrasound and plain films were 67.6%, 58.8% and 76.4%, respectively.

## Conclusion

Point-of care ultrasound may be a useful imaging modality for detecting proximal ureteral stone, but not for distal ureteral stone.

